# Influence of Ginger on Exercise-Induced Muscle Pain

**DOI:** 10.1093/nutrit/nuaf309

**Published:** 2026-05-26

**Authors:** Patrick J O’Connor

**Affiliations:** Department of Kinesiology, University of Georgia, Athens, GA 30602-6554, United States

**Keywords:** exercise, ginger, muscle, pain, physical activity, Zingiber officinale

## Abstract

The author investigated ginger’s potential effects on exercise-induced muscle pain. Initial experiments using a single 2-g dose of capsulized ginger yielded null results, leading to a shift to chronic supplementation experiments. After 11 continuous days of consuming 2 g of either raw or heat-treated ginger, delayed-onset muscle pain caused by eccentric exercise was reduced by 23%–25% compared with placebo. This research appears to have had a substantial impact, as evidenced, in part, by citation metrics as well as an associated increase in scientific and popular interest in ginger’s role in health and pain management.

## INTRODUCTION

At the start of the 21st century, the result of a pioneering experiment was reported that found that, compared with placebo, a twice-daily ginger extract significantly reduced knee pain in a sample of 247 patients with osteoarthritis.^1^ By 2006, at least 2 other smaller clinical trials also showed that ginger outperformed placebo in reducing pain in patients with osteoarthritis. Other studies revealed that ginger had anti-inflammatory effects in vitro (eg, by blocking biosynthesis of cyclooxygenase and leukotrienes) and could interfere with transient receptor potential vanilloid nociceptors (TRPV1) known to be involved in pain processing.^2^ This literature supported further research aimed at considering whether ginger also could attenuate muscle pain caused by exercise.

Given the large global health burden of musculoskeletal pain,^3^ it is unfortunate that muscle pain continues to be understudied. In 2006, exercise was under-studied as an experimental model to better understand human muscle pain. It is better known today that different types of exercise can systematically produce either (1) a noninflammatory acute muscle ischemia type pain or (2) minor muscle injury that activates inflammatory processes and results in delayed-onset muscle pain. The magnitude of noninflammatory muscle pain can be easily manipulated during a single session of exercise by varying the pedal resistance during leg cycling.^4^ Unaccustomed eccentric exercise (eg, lowering heavy dumbbells) can be used to cause (biceps) muscle injury, inflammation, and pain 24–72 hours postexercise, the magnitude of which can be controlled by manipulating the number of repetitions and/or the intensity of the exercise.^5^ Summarized below are 4 experiments that tested the effect of ginger on both of these types of muscle pain.

## METHODS AND RESULTS

### Acute Studies

Our first double-blind, crossover experiment examined the influence of consuming capsules containing a 2-g dose of ginger or flour placebo on ischemic quadriceps muscle pain during a single bout of moderate-intensity cycling exercise performed 45-minutes postconsumption by 25 university students.^6^ Compared with placebo, ginger had no meaningful effect on perceptions of induced quadriceps muscle pain or other measures performed during exercise, such as perceptions of effort or heart rate. Our second experiment was completed by participants who had not engaged in regular weight training of the biceps muscles during the prior 9 months. The experiment used a double-blind crossover design and tested the acute effect of a 2-g dose of ginger on arm (biceps) pain both 24 and 48 hours after performing eccentric exercise that induced inflammation (ie, arm swelling), arm dysfunction (ie, reduced range of motion and isometric force), and moderate-intensity arm pain.^7^ The investigation found that a single 2-g dose of ginger did not attenuate eccentric exercise–induced muscle pain, inflammation, or arm dysfunction 45 minutes after ingestion. With a half-life in humans of 1–3 hours,^8^ ginger metabolites are cleared and would be undetectable in humans 18 hours postconsumption. Participants refrained from ginger consumption for at least 48 hours prior to testing in these acute studies. Ginger metabolites are not stored in any tissues that were tested in response to the single dose of ginger. Participants consumed the capsules only after a nose-clip was placed to eliminate the smell of ginger.

We puzzled over these null findings, which could have resulted from multiple factors such as whether the timing of the ginger administration was optimal, if the active compounds in ginger became adequately bioavailable, and if a single 2-g dose was sufficient to produce a measurable change in pain. In the end, a decision was made to shift to a multiple-day dosing protocol and test if ginger taken chronically might have a demonstrable effect on pain.

### Chronic Studies

**Figure 1.**
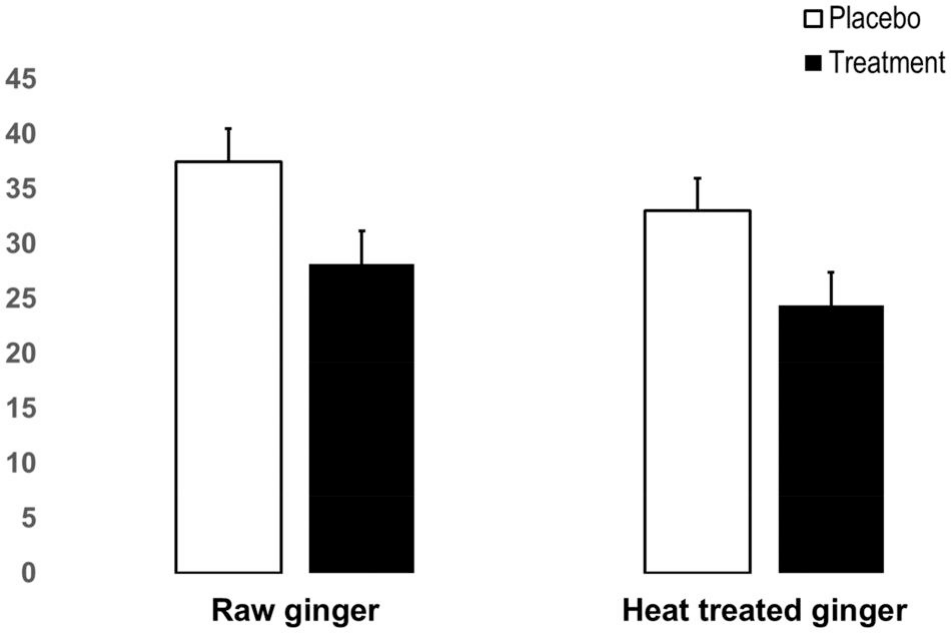
Mean (SE) Arm Muscle Pain Intensity Ratings (0–100 Visual Analog Scale [VAS]) 24 Hours After Eccentric Exercise in Placebo and Treatment Conditions Following 11 Consecutive Days of Consuming Capsules Containing 2 g of Either Raw Ginger or Heat-Treated Ginger. Pain intensity was reduced by an average of 9.3 and 8.6 VAS units, respectively. Figure created based on reference^9^.

The 2 chronic experiments tested the effects of 11 days of 2 g of raw (chronic study 1) or heat-treated (chronic study 2) ginger supplementation on muscle pain. Heat treating was used to increase the concentration of shogaols, a dehydrated form of gingerols. Shogaols more strongly activate TRPV1 nociceptors, which could lead to greater pain relief. The studies were identical double-blinded, placebo-controlled, randomized experiments. In both experiments, participants were required to report having not engaged in regular moderate-to-high-intensity weight training of the biceps brachii muscle during the previous 9 months. Participants were required to visit the laboratory each day and consume the capsules under supervision; the compliance was 100%. On the first testing day, the participants performed 18 eccentric actions of the nondominant elbow flexors to induce injury, inflammation, and pain. Compared with placebo, both raw and heat-treated ginger resulted in similar (23% to 25%) reductions in pain 24 hours after eccentric exercise.^9^ The pain intensity findings are illustrated in **Figure 1**.

At the end of the last day of testing, the participants indicated whether they had consumed ginger or placebo and how certain they were of this indication (0% to 100%). Of the 17 participants in the ginger group in chronic study 1, those who guessed their condition correctly and reported a high certainty (>50% certainty) of having consumed ginger (*n* = 10) did not have lower pain-intensity ratings 24 hours after exercise than those who were uncertain about whether they had consumed ginger (*n* = 7) (mean pain of 27.1 mm vs 28.0 mm, respectively and based on the 0–100 Visual Analogue Scale). In study 2, none of the 8 participants who guessed their condition correctly (>50% correct) reported a high certainty of having consumed ginger (ie, ≤51% certainty). These data discount the possibility that ineffective blinding accounted for the results.

## DISCUSSON

One subsequent placebo-controlled experiment conducted by other researchers found no effect of 5 days of 4 g of ginger supplementation on delayed-onset muscle pain.^10^ We decided on 11 continuous days of ginger consumption in order to balance the risks of overburdening the study participants and potentially reducing study compliance if we had used 2 or more weeks of ginger consumption against striving to ensure enough days/time for the accumulation of ginger bioactives in neural tissue—which was an unknown. However, the pain-reducing effects of ginger observed in our 2 studies may have underestimated the true effect of ginger because, to maximize the double-blinding procedure, we had participants consume the capsules while wearing a nose clip that prevented them from smelling any ginger aromatics. Others have observed psychological effects from ginger olfaction. For example, nasal skin application of the essential oil of ginger is associated with a 20% reduction in nausea following surgery involving anesthesia.^11^ Not all potential confounders could be controlled—for example, nutritional status before, and diet across, the study was not assessed. However, we did show that the eccentric exercise reduced isometric muscle force and range of motion while increasing arm volume—indicators that muscle damage occurred.

**Figure 2.**
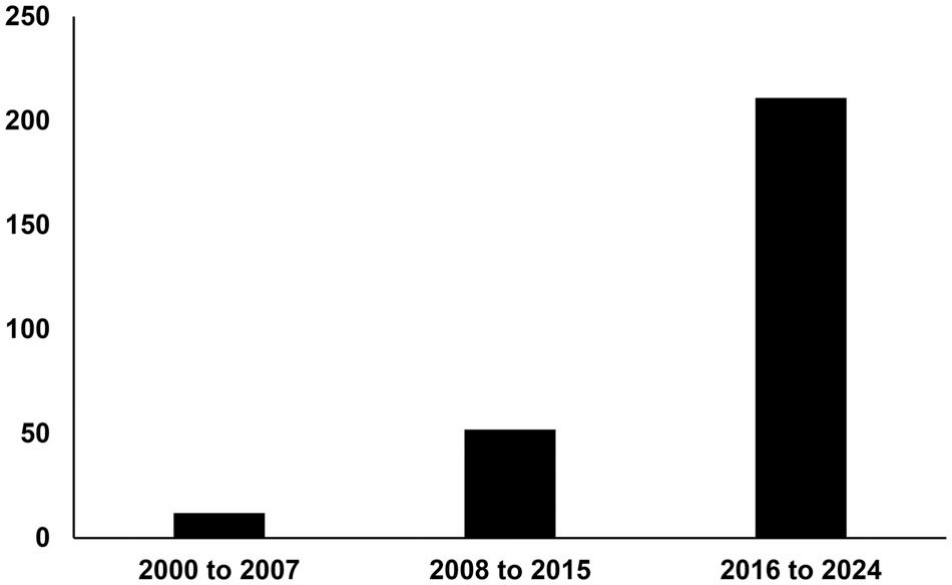
Number of Published Research Papers Using the Words “Ginger” and “Pain” in the Title Since the Year 2000. Searches were conducted by the author using the words ginger and pain on February 17, 2025, and using the advanced “in the title” search option in Google Scholar.

The impact of research can be difficult to document in clear and precise ways. Keeping that caveat in mind, one potential measure of impact is that the 3 articles that summarized the 4 research projects on ginger and exercise-induced pain have been well cited by other scientists. The null findings for the acute effects of ginger on cycling pain and delayed inflammatory pain have been cited 77 and 61 times, respectively, based on a Google Scholar search (https://scholar.google.com). According to 1 metric, this demonstrates a moderate influence in the research community.^12^ Our study showing that 11 days of raw or heat-treated ginger supplementation reduces muscle pain caused by eccentric exercise has been cited 340 times, and it has been suggested that this number of citations provides evidence of a broad and influential impact on scientists.^13^ Other potential measures of impact include that, in the years following publication of the research, there has been (1) an increased number of research papers with both the words “ginger” and “pain” in the title (see **Figure 2**) and (2) an increased worldwide research interest in ginger and health.^14–16^ In sum, there are multiple potential factors for the increased interest in ginger and it is plausible that the investment in research into the health consequences of ginger is 1 reason.

## CONCLUSION

Chronic consumption of at least 2 g of ginger for at least 11 days reduces delayed-onset muscle pain intensity caused by eccentric exercise by a meaningful amount compared with placebo.

## Data Availability

No new data was included in this review article except the Google Scholar search results presented in Figure 2. Interested readers can use the information provided in the Figure legend to reproduce the data shown.
